# A Case of an Atraumatic Posterior Perirenal Lumbar Hernia

**DOI:** 10.7759/cureus.33793

**Published:** 2023-01-15

**Authors:** Kristin N Slater, Moustapha Doulaye, Uzoamaka Obodo, Ann George, Adnan Mohammadbhoy

**Affiliations:** 1 Dermatology, Lincoln Memorial University DeBusk College of Osteopathic Medicine, Harrogate, USA; 2 Surgery, Bravera Health Seven Rivers, Crystal River, USA; 3 Internal Medicine, Lincoln Memorial University DeBusk College of Osteopathic Medicine, Harrogate, USA; 4 General Surgery, Bravera Health Seven Rivers, Crystal River, USA

**Keywords:** atraumatic hernia, lumbar hernia, primary hernia, perirenal hernia, perirenal, primary lumbar hernia

## Abstract

Retroperitoneal lumbar hernias are a rare entity. Atraumatic posterior perirenal hernias are an exceptionally rare form of retroperitoneal lumbar hernias. Because of their infrequency, there are no standardized methods of surgical care for the treatment of atraumatic (primary spontaneous) posterior perirenal hernias. This report documents the finding and management of an atraumatic posterior perirenal lumbar hernia in a 69-year-old female.

## Introduction

Retroperitoneal lumbar hernias are rare and consequentially have limited standardized treatment recommendations [1]. Posterior perirenal hernias are a form of lumbar hernia that can be related to surgical procedures [2-4] or can be primary, i.e., spontaneous and unrelated to surgery [4]. Incisional hernias have been noted in post-renal transplants [2], retroperitoneal robotic partial nephrectomies [3], and general renal surgeries [4]. In large defects where the kidney is involved, a supportive mesh has been used to repair the defect [5-6]. Contributing risk factors in developing hernias after surgery include, but are not limited to, high body mass index (BMI), the use of self-retaining retractors, and infections [4]. In primary lumbar hernias, risk factors were reported to be related to increased intra-abdominal pressure and a weak posterior abdominal wall [7]. In a small study of lumbar hernia cases, it was reported that 24 out of 78 (30.8%) patients presented with incarcerated hernias [7]. Because of their high risk of incarceration, lumbar hernias are important to repair [7].

## Case presentation

**Figure 1:**
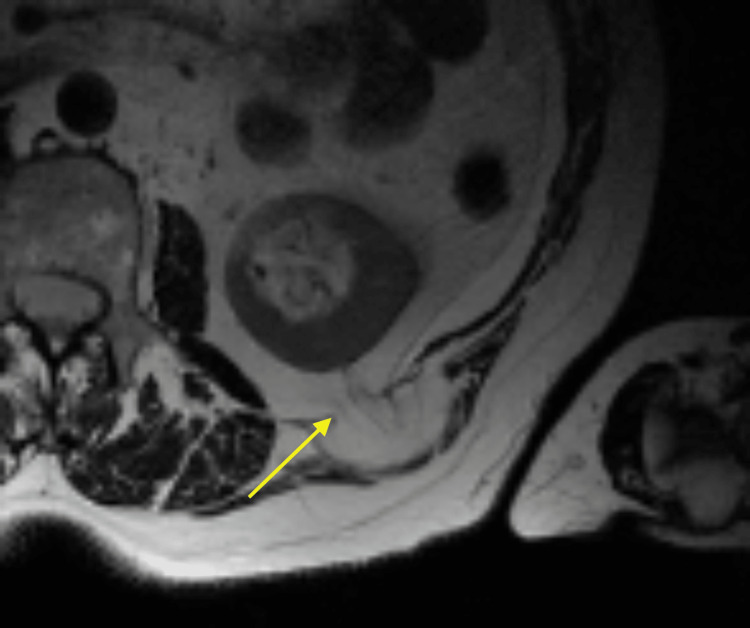
CT with an arrow showing a posterior perirenal hernia defect

**Figure 2:**
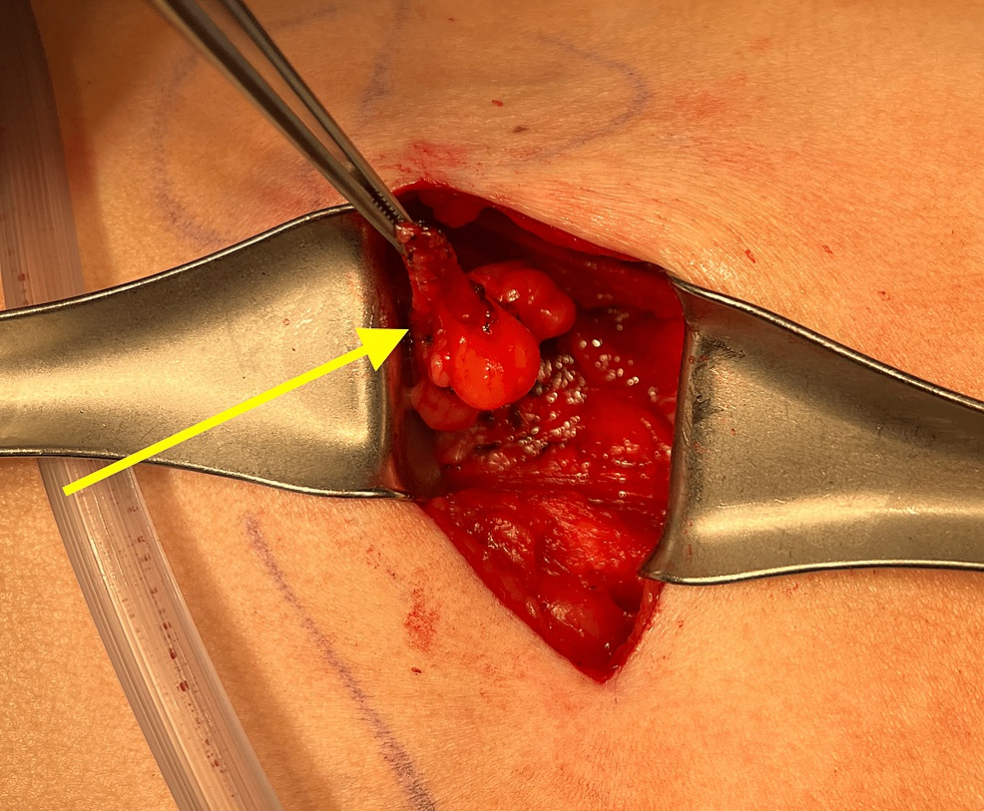
Intraoperative view of the hernia sac denoted by the yellow arrow

**Figure 3:**
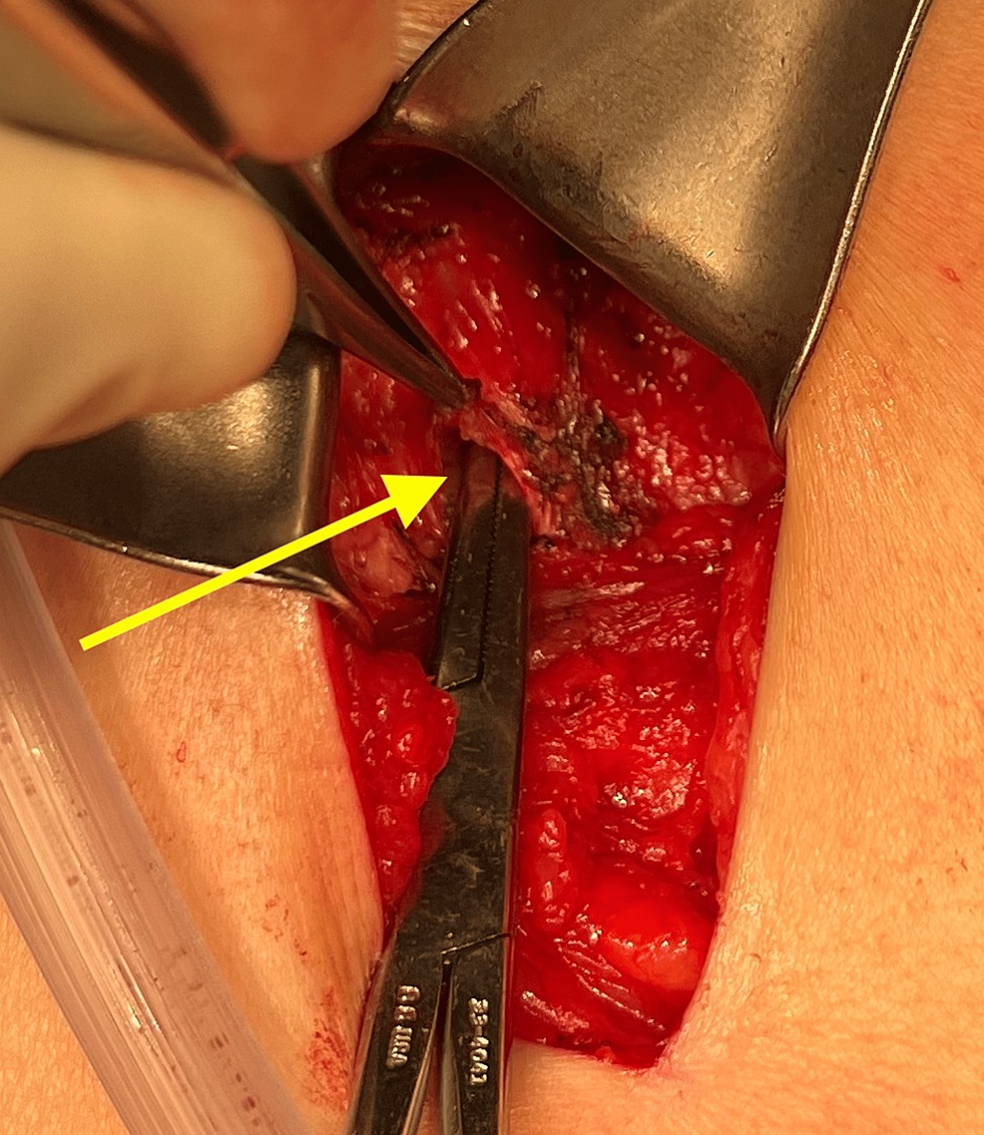
Intraoperative view of the hernia defect denoted by the yellow arrow

**Figure 4:**
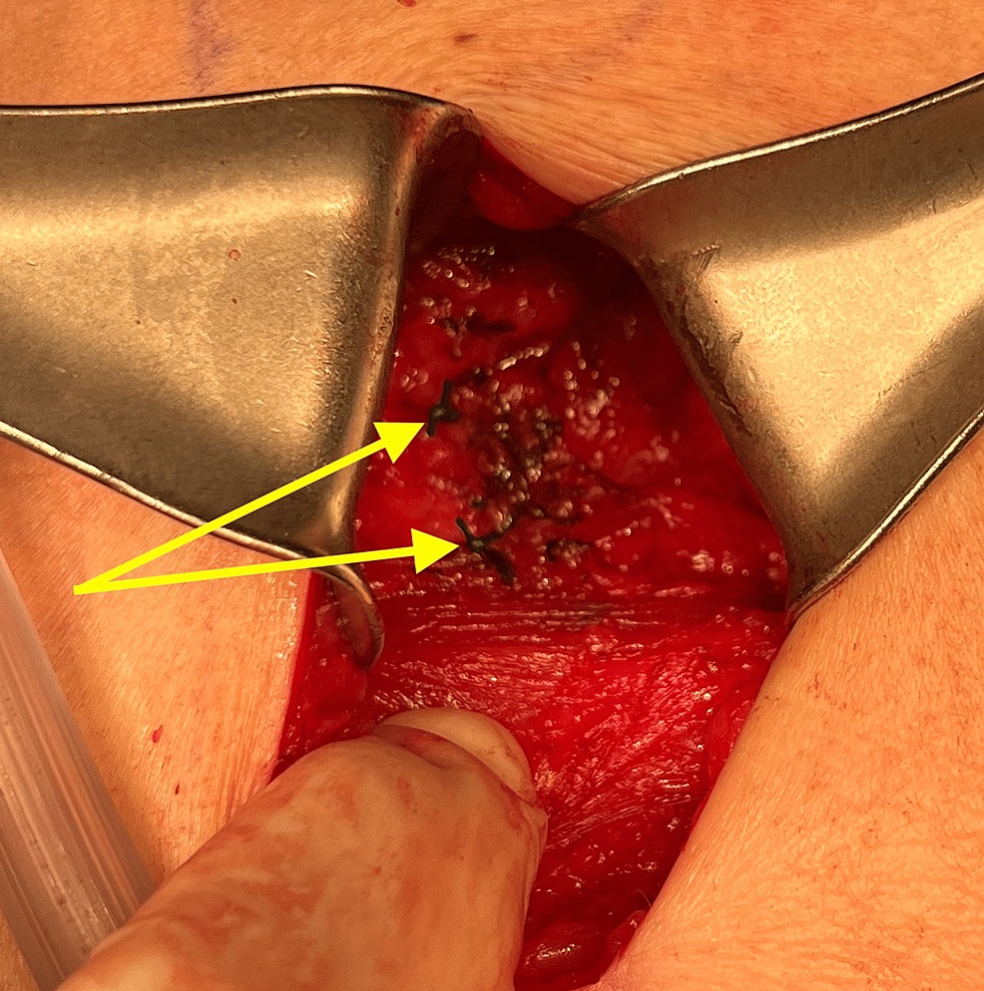
Closure of the hernia defect with a figure-of-eight suture; the yellow arrows denote suture tails

**Figure 5:**
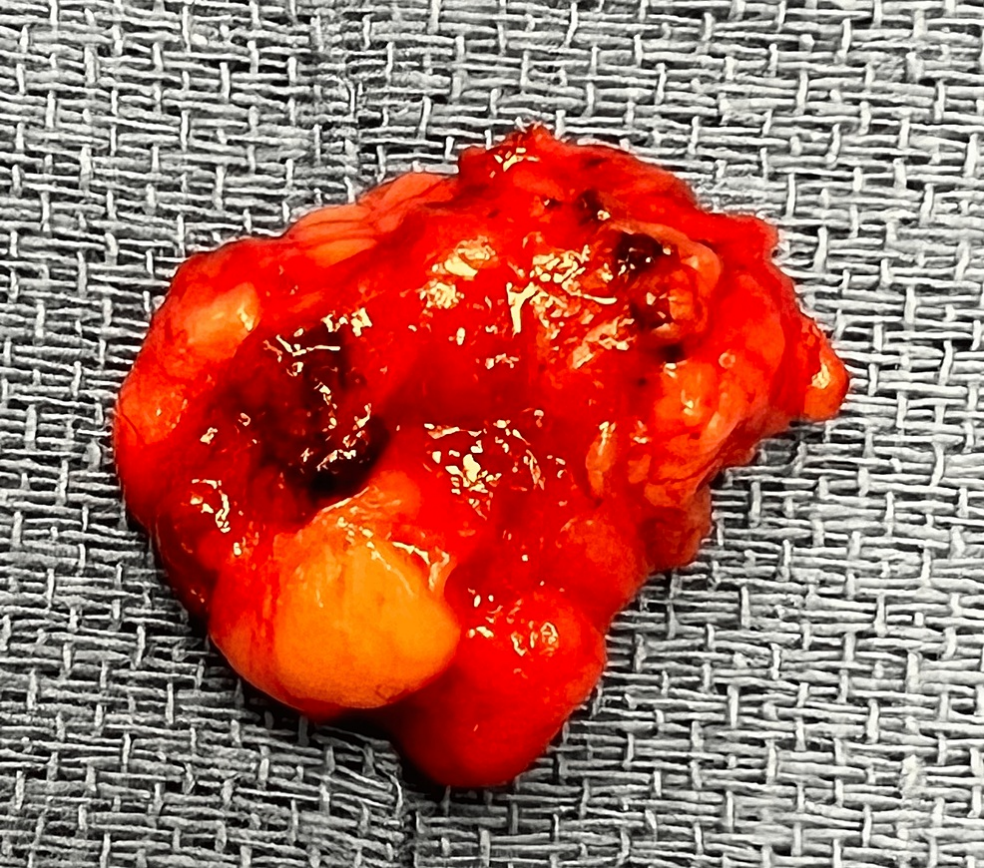
Gross pathology of the hernia sac

A 69-year-old female presented to the office with the chief complaint of a soft tissue mass on the left side of the posterior lateral back. Her medical history included hypertension, asthma, depression, hypothyroidism (secondary to radioactive iodine treatment), and osteoporosis. Her BMI was 25.84, and her medications included vitamin D3, tramadol hydrochloride (HCL), simvastatin, sertraline HCL, denosumab, pantoprazole sodium, lisinopril-hydrochlorothiazide, levothyroxine, and cholestyramine. Surgical history was significant for cholecystectomy, bladder suspension, hysterectomy, oophorectomy, and femoral hernia repair. The mass began weeks prior and had been increasing in size. Pressure was noted to aggravate the area, contributing to pain, especially on prolonged sitting. She had no history of trauma or surgery in the area. Clinically, a solitary mass measuring approximately 5 cm underlying unremarkable-appearing skin was appreciated. Due to the depth of the mass and concern for a potential sarcoma or another tumor, magnetic resonance imaging (MRI) was ordered, which showed a fat hernia from the left perirenal space through the left flank of the posterior lateral abdominal wall most consistent with a Grynfeltt type hernia of the superior lumbar triangle (Figure 1). The hernia measured 4 cm x 2 cm with a narrow neck measuring 8 mm in diameter. The results were discussed with the patient, who ultimately opted for an open retroperitoneal lumbar hernia repair. Intraoperatively, an incision was made to the hernia sac, which was dissected circumferentially down to the level of the fascia (Figure 2). This was opened and then excised at the level of the retrorenal fascia. The hernia defect remained small, and no mesh was required for repair (Figure 3). The fascia was approximated using a figure-of-eight suture (Figure 4). There was good approximation and coverage. The surgical site was closed in layers with 3-0 vicryl and 4-0 monocryl and then dressed with 2-octyl cyanoacrylate. The gross pathology exhibited a portion of fibromembranous adipose tissue measuring 3 cm in diameter (Figure 5). The pathology report, as expected, showed a left retroperitoneal hernia sac.

## Discussion

Lumbar hernias are a rare finding [1], requiring surgical attention because of the high risk of incarceration and potential risk of strangulation [7]. Lumbar hernias can be acquired secondary to surgical procedures [2-4] or they can be primarily acquired [8]. Primarily acquired (spontaneous/atraumatic) lumbar hernias represent 55% of all lumbar hernias [8]. Risk factors for primary lumbar hernias were reported to be related to increased intra-abdominal pressure and posterior abdominal wall weakness [7]. Increased age was also reported to be a risk factor in acquiring lumbar hernias [4,7]. Some reports have noted no significant difference in post-operative lumbar hernias based on sex, but the reports are inconsistent [4]. Lumbar hernias can be insidiously difficult to diagnose and easy to misdiagnose, as they can be asymptomatic and/or non-palpable [7], and in some cases, only mildly uncomfortable [8]. It is important to complete a thorough workup to avoid a misdiagnosis of a subcutaneous lesion such as a lipoma [6,8-9]. MRI or CT are the best methods for diagnosis, with a preference for CT scans [7-9]. In our case, an MRI was used due to the concern of a potential sarcoma or another type of tumor. Like other hernias, lumbar hernias tend to increase in size over time making surgical intervention the favorable option [7]. If renal incarceration or strangulation occurs, it can be serious, making the option of elective surgical intervention preferred [7]. Because they are underrepresented in the literature, there are no standardized recommendations for treatment [1]. In our case, the defect was small, and a primary repair was attainable without mesh, although in larger incisional herniations or herniations with kidney involvement, a mesh has been used to reinforce the site [5-6]. In our case, no incisional hernia was noted on the patient's follow-up after surgery. We will continue to monitor the patient for the development of an incisional hernia. More representation in the literature and further research would be beneficial to better standardize the diagnosis and treatment of posterior perirenal lumbar hernias.

## Conclusions

In the rare cases of posterior perirenal lumbar hernias, it is appropriate to surgically intervene due to the risk of renal herniation and the potential risk of strangulation. Our case presented an atraumatic posterior perirenal lumbar hernia containing perirenal fat with a small defect that was able to be primarily repaired without mesh. Our patient did well postoperatively and will continue to follow up for monitoring of the incision site. Further categorization and standardization of lumbar hernias and corresponding repair techniques would be a useful area of further research.

## References

[1] Beffa LR, Margiotta AL, Carbonell AM (2018). Flank and lumbar hernia repair. Surg Clin North Am.

[2] Moris D, Bokos J, Vailas M (2017). Renal paratransplant hernia revealed: a review of the literature. Hernia.

[3] Chow AK, Wahba BM, Phillips T (2021). Incisional lumbodorsal hernias following retroperitoneal robotic partial nephrectomies for small renal masses at a high-volume tertiary referral center. J Endourol.

[4] Osman T, Emam A, Farouk A, ElSaeed K, Tawfeek AM, AbuHalima A (2018). Risk factors for the development of flank hernias and bulges following surgical flank approaches to the kidney in adults. Arab J Urol.

[5] Zatloukal A (2020). Lumbar hernia - case report. Rozhl Chir.

[6] Mehrabi S, Yavari Barhaghtalab MJ, Babapour M (2020). Renal pelvis and ureteropelvic junction incarceration in a Grynfeltt-Lesshaft hernia: a case report and review of the literature. BMC Urol.

[7] van Steensel S, Bloemen A, van den Hil LC, van den Bos J, Kleinrensink GJ, Bouvy ND (2019). Pitfalls and clinical recommendations for the primary lumbar hernia based on a systematic review of the literature. Hernia.

[8] Kadler B, Shetye A, Patten DK, Al-Nowfal A (2019). A primary inferior lumbar hernia misdiagnosed as a lipoma. Ann R Coll Surg Engl.

[9] Heo TG (2021). Primary Grynfeltt's hernia combined with intermuscular lipoma: a case report. Int J Surg Case Rep.

